# Low Seroprevalent Species D Adenovirus Vectors as Influenza Vaccines

**DOI:** 10.1371/journal.pone.0073313

**Published:** 2013-08-22

**Authors:** Eric A. Weaver, Michael A. Barry

**Affiliations:** 1 Division of Infectious Diseases, Mayo Clinic, Rochester, Minnesota, United States of America; 2 Department of Immunology, Mayo Clinic, Rochester, Minnesota, United States of America; 3 Department of Molecular Medicine, Mayo Clinic, Rochester, Minnesota, United States of America; 4 Translational Immunovirology and Biodefense Program, Mayo Clinic, Rochester, Minnesota, United States of America; Instituto Butantan, Brazil

## Abstract

Seasonal and pandemic influenza remains a constant threat. While standard influenza vaccines have great utility, the need for improved vaccine technologies have been brought to light by the 2009 swine flu pandemic, highly pathogenic avian influenza infections, and the most recent early and widespread influenza activity. Species C adenoviruses based on serotype 5 (AD5) are potent vehicles for gene-based vaccination. While potent, most humans are already immune to this virus. In this study, low seroprevalent species D adenoviruses Ad26, 28, and 48 were cloned and modified to express the influenza virus A/PR/8/34 hemagglutinin gene for vaccine studies. When studied *in vivo*, these species D Ad vectors performed quite differently as compared to species C Ad vectors depending on the route of immunization. By intramuscular injection, species D vaccines were markedly weaker than species C vaccines. In contrast, the species D vaccines were equally efficient as species C when delivered mucosally by the intranasal route. Intranasal adenovirus vaccine doses as low as 10^8^ virus particles per mouse induced complete protection against a stringent lethal challenge dose of influenza. These data support translation of species D adenoviruses as mucosal vaccines and highlight the fundamental effects of differences in virus tropism on vaccine applications.

## Introduction

Influenza virus infections impose a significant burden on our society. Annually, 5-15% of the world’s population is affected by epidemics and have upper respiratory tract infections, 3 to 5 million have severe illness and 250,000 to 500,000 cases result in death [1]. In the U.S.A. seasonal influenza affects up to 20% of the population, results in 200,000 hospitalizations and approximately 37,000 deaths each year. The World Health Organization (WHO) states, "Influenza rapidly spreads around the world in seasonal epidemics and imposes a considerable economic burden in the form of hospital and other health care costs and lost productivity. In the United States of America, it is estimated that influenza epidemics cost up to $167 billion per year [2]”.

While these seasonal epidemics are certainly of concern, pandemic influenza outbreaks are of substantially higher concern. During the past century there have been several severe pandemics [1]. In 1918-1919, an H1N1 known as the Spanish Flu caused the world’s largest influenza pandemic killing 20-40 million people. In 1957, the Asian flu caused by an H2N2 virus resulted in ~1.5 million deaths and in 1968, the Hong Kong flu caused by an H3N2 influenza resulted in ~1 million deaths.

Trivalent Inactivated Vaccine (TIV) is the standard for influenza vaccination and has protected millions of humans from influenza morbidity and mortality. While somewhat effective, TIV has limitations that support the development of alternate vaccine platforms. Some of the limitations to using TIV include the following: The use of embryonated eggs is a fundamental problem with this vaccine platform. A large fraction of humans are allergic to egg ovalbumin and cannot take this vaccine. Generating influenza vaccines in eggs is time consuming and labor intensive and relies on the availability of embryonated eggs. TIV only provides short-term immunity [3] and this immunity is highly strain specific [4,5]. Intramuscular delivery does not stimulate high levels of the secretory IgA that is thought to be more reactive against heterologous viruses (12,26,27,31,38). Importantly, the TIV vaccine also fails to induce cross-protective cellular immunity [5–9]. Finally, one of the most fundamental problem relates to the requirement that human influenza virus be adapted to grow in chicken eggs before a vaccine can be produced [10–14]. A perfect example of this problem was during the 2009 H1N1 swine flu pandemic. In this case, delivery of the 2009 monovalent inactivated vaccine (MIV) was delayed by months due to slow growth of this virus during egg-based vaccine production [15]. In addition to these limitations a recent study of the TIV vaccine efficacy showed that it was only effective 59% of the time [16].

FluMist is a live-attenuated cold-adapted influenza virus platform that is administered intranasally. While this new platform has the advantage of being needle-free and induces a low level of cellular immunity it still has many limitations that support the study of alternative vaccine platforms. These limitations include cost, egg allergies, the vaccine is restricted to certain age groups, priming requires two doses of vaccine, it cannot be administered to immunocompromised patients, and, importantly, studies have shown that the protective immune responses induced by Flumist decline more rapidly than the TIV [17–19] indicating a need for new influenza vaccine technologies.

Adenoviruses are non-enveloped linear double-stranded DNA viruses. Human adenoviruses have been classified into 57 unique types that are defined into 7 species (A-G) [20]. Adenoviruses are attractive viral vectors for use as vaccines, since viral genes can be readily removed and replaced with antigen genes from a pathogen.

One of the most studied and used Ad vectors was derived from the species C adenovirus type 5 (Ad5). Unfortunately, epidemiological studies show that Ad5 is one of the most seroprevalent of these human viruses and is therefore more likely to be less effective in human vaccinees [21–23]. Beyond Ad5, there are a number of human adenoviruses that are markedly less seroprevalent in the population. Given that these are less likely to be neutralized in humans during vaccination, we and others have tested them as alternatives to Ad5 [24]. In this work, we cloned the genomes from three low seroprevalent species D adenoviruses [21,25,26] and modified these to carry antigens from influenza. We have compared these vaccines to the Ad5 benchmark vaccine by intramuscular and intranasal routes of vaccination to protect mice from a stringent lethal challenge by influenza virus.

## Results

### Protection Against Heterologous Influenza by Traditional Influenza Vaccine Versus Ad5 Gene-based Vaccine

Trivalent Inactivated Vaccine (TIV) is a protein-based vaccine and is the standard for seasonal influenza vaccination. The 2009 monovalent inactivated vaccine (MIV) is also a protein-based vaccine, but in this case directed against only one strain of influenza (A/CA/07/09) [27].

To assess the utility of Ad vectored vaccines against influenza, we compared a standard replication-defective Ad5 (Ad5-RD) expressing the HA from the 2009 H1N1 swine flu to a traditional protein-based MIV vaccine. In 2009-2010, human vaccinees received 15 µg doses of the 2009 MIV by the i.m. route. For a 70 kg human, this corresponds to approximately 0.2 µg/kg doses. Mice were immunized with 4, 40, or 1,500 ng of MIV by the i.m. route, which corresponds approximately to 0.2, 2.0, and 75 µg/kg doses. Parallel groups of mice were immunized with 10-fold dilutions of Ad5 expressing codon-optimized hemagglutinin (HA) from a 2009 H1N1 strain A/TX/05/09 (Ad5-TX). The MIV and Ad5-TX vaccines both contain isolates from the 2009 swine flu pandemic. The HA protein sequences of these two isolates are 99.5% identical. The doses ranged from 10^10^-10^6^ virus particles (vp)/mouse. These doses correspond to delivery of 0.75 to 7500 ng of Ad protein. It should be noted, that this protein concentration is made up of Ad virus proteins and not influenza protein antigens, but is provided simply for comparison to the MIV vaccine.

**Figure 1:**
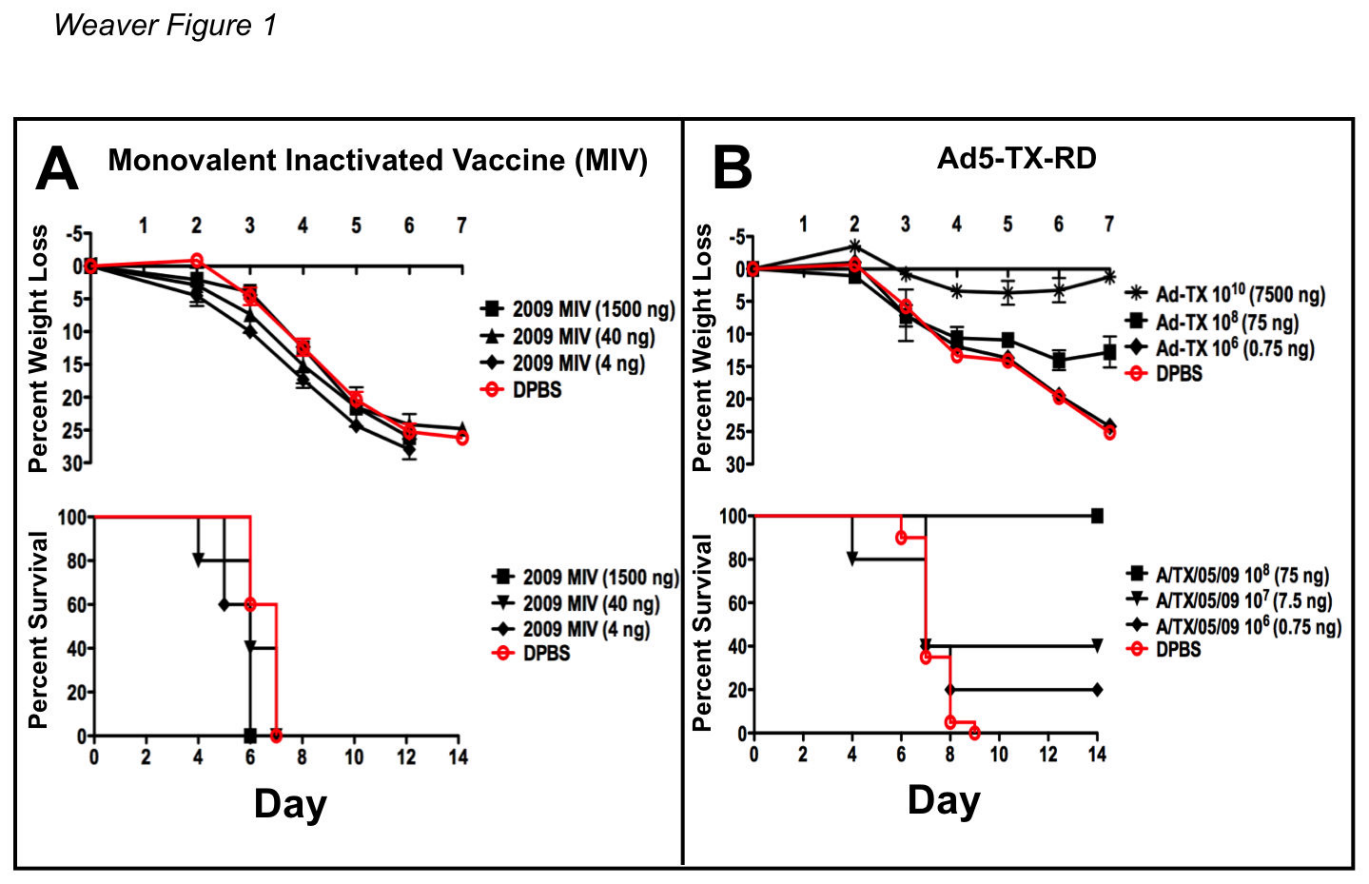
Comparison of cross-protection by monovalent 2009 H1N1 vaccine (MIV) vs. Ad expressing 2009 H1N1 HA (Ad-TX). Groups of 5 BALB/c mice were immunized intramuscularly with either the MIV (A) or the Ad-TX vectored vaccine (B) at the indicated vaccine doses and then challenged intranasally with A/PR/8/34. Weight loss and survival was monitored. There were no cross-protective immune responses induced in the MIV immunized mice and all mice were sacrificed due to weight loss by day 7. However, 100% of mice immunized with ≥10^8^ viral particles (75 ng) of Ad-TX had cross-protective immune responses and were completely protected from death.

BALB/c mice were immunized intramuscularly with varying doses of the two vaccines in order to titrate the level of protection induced by the two vaccine platforms (Figure 1). The mice were then challenged intranasally 3 weeks later with 100 MLD_50_ of mouse-adapted influenza A/PR/8/34 to model a virus challenge heterologous to both of the vaccines. Under these conditions, the MIV vaccine did not provide any detectable cross-protection against A/PR/8/34 as assessed by weight loss and survival (Figure 1A). In contrast, the Ad5-TX vaccine reduced weight loss at doses ≥ 10^8^ vp/mouse and increased survival at all doses (Figure 1B). A dose as low as 10^8^ vp/mouse of Ad5-TX resulted in 100% survival as compared to 0% survival at the highest dose of MIV. Higher doses of Ad5-TX (10^10^) protected mice completely against both weight loss and death. Cross protection by Ad expressed HA genes had been observed previously. Ad5 expressing full-length A/PR/8/34 HA was shown to provide cross-protection against A/FM/1/47 and A/CA/04/09 [28].

### Generation of Lower Seroprevalent Ad Vaccines against Influenza

Ad5 is a potent vaccine vector. However, most humans are immune to the virus and this immunity would likely blunt vaccine efficacy. Given this, we cloned the genome for Ad6. Ad6 is a lower seroprevalent species C virus (Figure S1) similar to Ad5 [29]. We also cloned three low seroprevalent species D viruses, Ad26, 28, and 48 (Figures S2-S4). These viruses not only differ in terms of human immunity, but vary in tropism. Species C viruses use the coxsackie and adenovirus receptor (CAR) and **α**v integrins and species D viruses use integrins, CD46, and/or sialic acid as receptors for virus entry. Each viral genome was cloned and replication-competent or replication-defective Ad vaccines were constructed by replacement of the E3 or E1 regions with an expression cassettes for the influenza hemagglutinin (HA) gene.

### Comparison of Immunity Against Influenza Induced By Species C And D Ad Vaccines by Intramuscular Immunization

**Figure 2:**
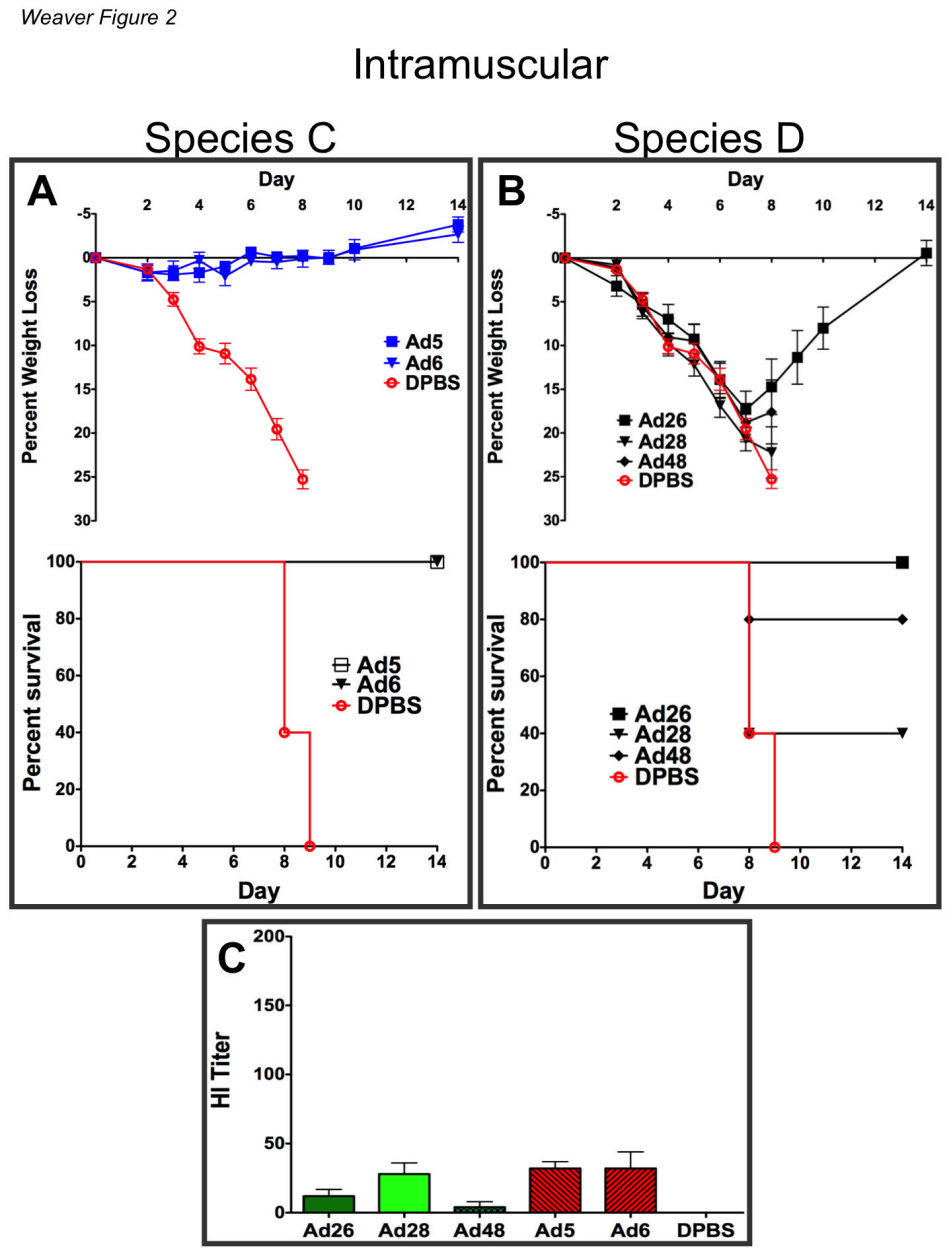
Vaccine efficacy of species C and D Ad vectors by high dose systemic immunization. Groups of 5 BALB/c mice were administered 10^10^ viral particles of the indicated vectors intramuscularly. All viral vectors contained a transgene cassette that expressed a soluble hemagglutinin (HA) derived from the influenza strain A/PR/8/34. Three weeks after immunization the mice were anesthetized i.p. with ketamine (140 mg/kg)/xylazine (5.55 mg/kg). The mice were challenged intranasally with 100MLD_50_ of influenza A/PR/8/34. The mice were weighed for baseline measurements. Included in the sacrifice criteria was 25% body weight loss.

We compared the two species C vaccines (Ad5 and Ad6) to three species D vaccines (Ad26, Ad28 and Ad48) as replication-competent vectors with intact E1 genes and HA replacing the E3 domain. An *in vitro* analysis of HA expression by the vectors showed that HA was expressed in a similar amount regardless of Adenovirus type or location of the transgene (Figure S7). BALB/c mice were immunized intramuscularly with 10^10^ vp/mouse of the species C and D Ad vaccines. Three weeks after immunization the mice were challenged intranasally with 100 MLD_50_ of influenza A/PR/8/34 (Figure 2). Under these conditions, both of the species C Ad vaccines mediated significant reductions in weight loss and 100% survival (Figure 2A). In contrast, species D Ad28 provided little protection (Figure 2B). Ad48 induced greater levels of protection than Ad28. However, only Ad26 vaccine mediated 100% survival. Even though Ad26 mice survived, there was still significant weight loss and disease in the animals as compared to Ad5 and Ad6. Statistical analyses showed that the Ad28 vaccinated mice had significantly higher mortality rate than either species C Ad vaccines (p = <0.05). A hemagglutination inhibition (HI) assay on sera from i.m. immunized mice showed weaker anti-influenza immune responses in species D Ad vaccinated mice (Figure 2C). Statistical analyses showed that both Ad5 and Ad6 induced significantly higher HI titers than Ad48 (p = <0.05).

### Comparison of Species C And D Ad Vaccines By Intranasal Immunization

**Figure 3:**
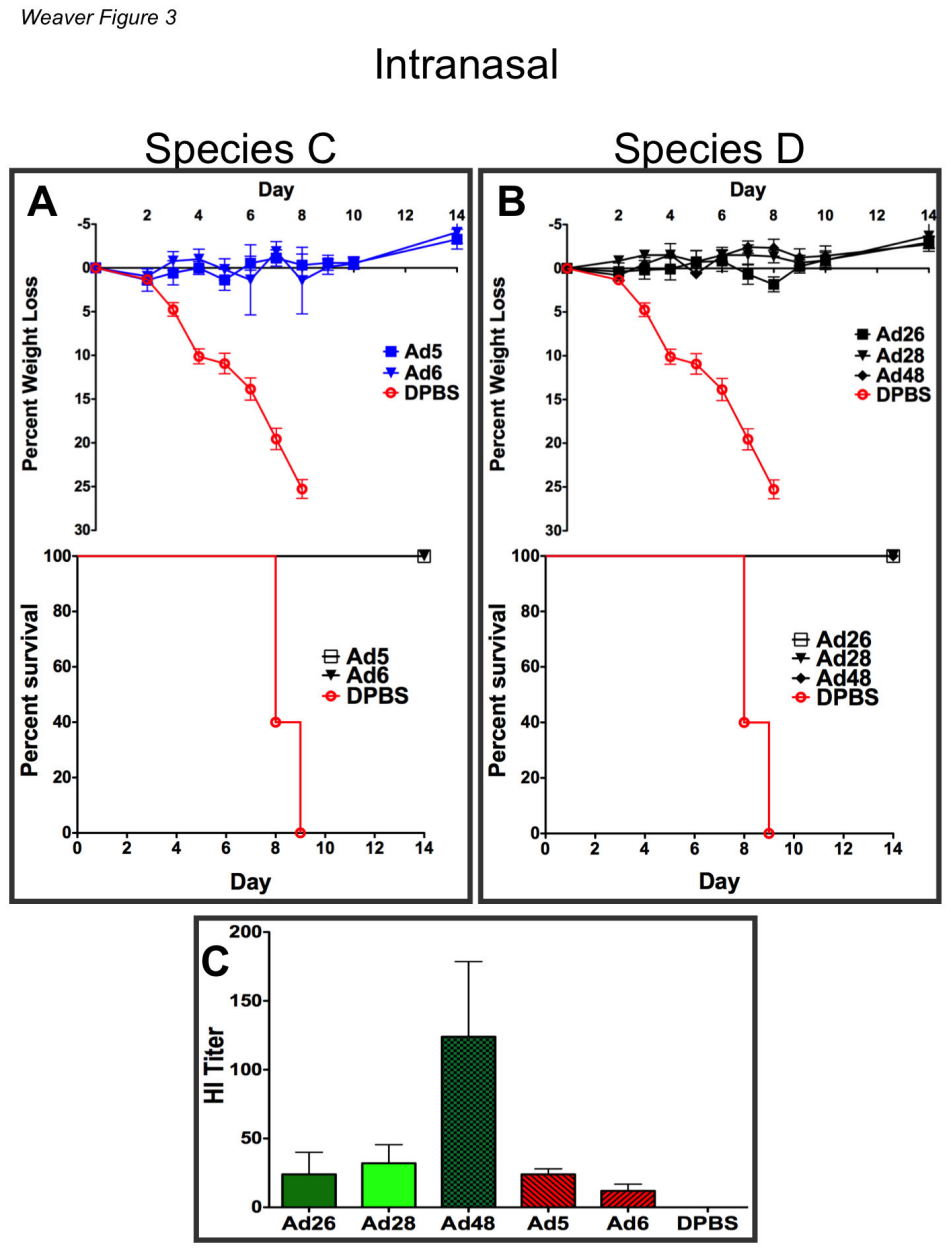
Vaccine efficacy of species C and D Ad vectors by high dose mucosal immunization. Groups of 5 BALB/c mice were administered 10^10^ viral particles of the indicated vectors intranasally. All viral vectors contained a transgene cassette that expressed a soluble hemagglutinin (HA) derived from the influenza strain A/PR/8/34. Three weeks after immunization the mice were anesthetized i.p. with ketamine (140 mg/kg)/xylazine (5.55 mg/kg). The mice were challenged intranasally with 100MLD_50_ of influenza A/PR/8/34. The mice were weighed for baseline measurements. Included in the sacrifice criteria was 25% body weight loss.

BALB/c mice were immunized intranasally with 10^10^ vp/mouse. This route stimulates the mucosa in a manner similar to that of the licensed FluMist vaccine. Three weeks after immunization the mice were challenged intranasally with 100MLD_50_ of influenza A/PR/8/34 (Figure 3). In contrast to the weak effects observed after i.m. immunization, the species D vaccines were as potent as the species C vaccines in protecting against influenza when delivered intranasally (Figure 3A and B). There were no detectable differences in protection induced by either the species C or D Ad vaccines at this dose and both species of Ad vaccines protected 100% of animals against death. In addition, both species C and D Ad vaccines prevented influenza-induced weight loss and disease. A HI assay on sera from mice immunized intranasally showed higher HI antibody titers in species D vaccinated mice (Figure 3C). In this case, Ad48 had statistically higher HI titers than both Ad5 and Ad6 (p = <0.05).

### Dose Titration Of Replication-competent Species C And D Vaccines

**Figure 4:**
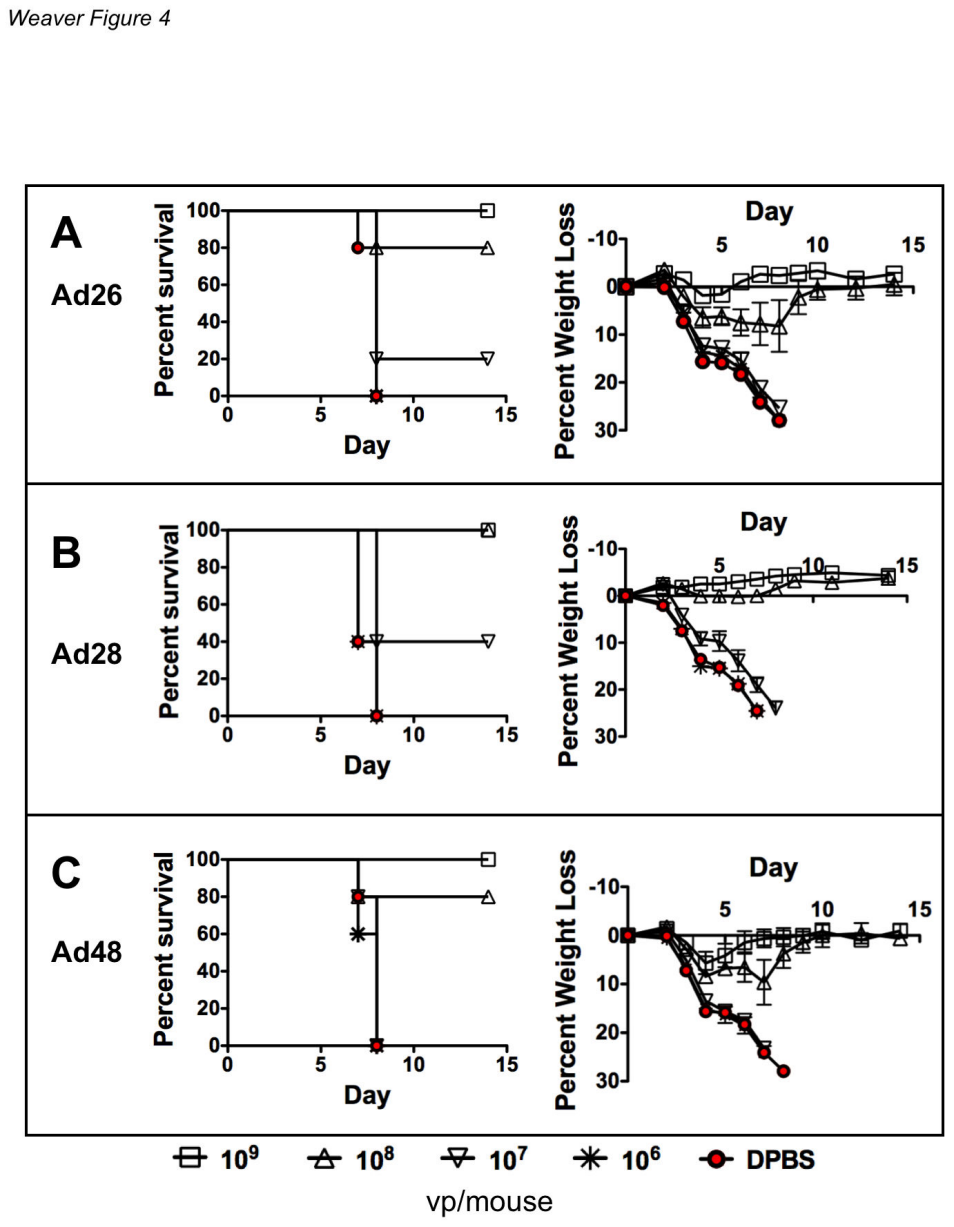
Dose titration of species D replication-competent Ad vectored vaccines. Groups of 5 BALB/c mice were immunized intranasally with 10-fold dilutions of vaccine from 10^9^ vp/mouse to 10^6^ vp/mouse. All viral vectors contained a transgene cassette that expressed a soluble hemagglutinin (HA) derived from the influenza strain A/PR/8/34 in place of the E3 genes. Mice were immunized with either species D Ad26 (A), Ad28 (B) or Ad48 (C). Three weeks after immunization the mice were anesthetized i.p. with ketamine (140 mg/kg)/xylazine (5.55 mg/kg). The mice were challenged intranasally with 100MLD_50_ of influenza A/PR/8/34. The mice were weighed for baseline measurements. Included in the sacrifice criteria was 25% body weight loss.

The initial vaccine comparisons used high doses of 10^10^ vp of the adenoviruses (Figures 2 and 3). To better determine the relative efficacy of the vaccines by i.n. immunization, a dose titration of each of the vaccines was performed. BALB/c mice vaccinated with 10^9^ vp/mouse showed no signs of weight loss and had 100% survival (Figure 4). Lower doses of 10^7^ vp/mouse allowed weight loss and death in all vaccinated groups. No protection was observed doses of 10^6^ vp/mouse (Figure 4). The Ad28 vaccine was the most effective of the species D vaccines and provided complete protection against disease and death at a dose of 10^8^ vp/mouse (Figure 4B). Ad26 and Ad48 vaccinated mice showed lost weight and only 80% survived at a dose of 10^8^ vp/mouse (Figure 4 A and C).

### Dose Titration of Replication-defective Ad Vaccines

**Figure 5:**
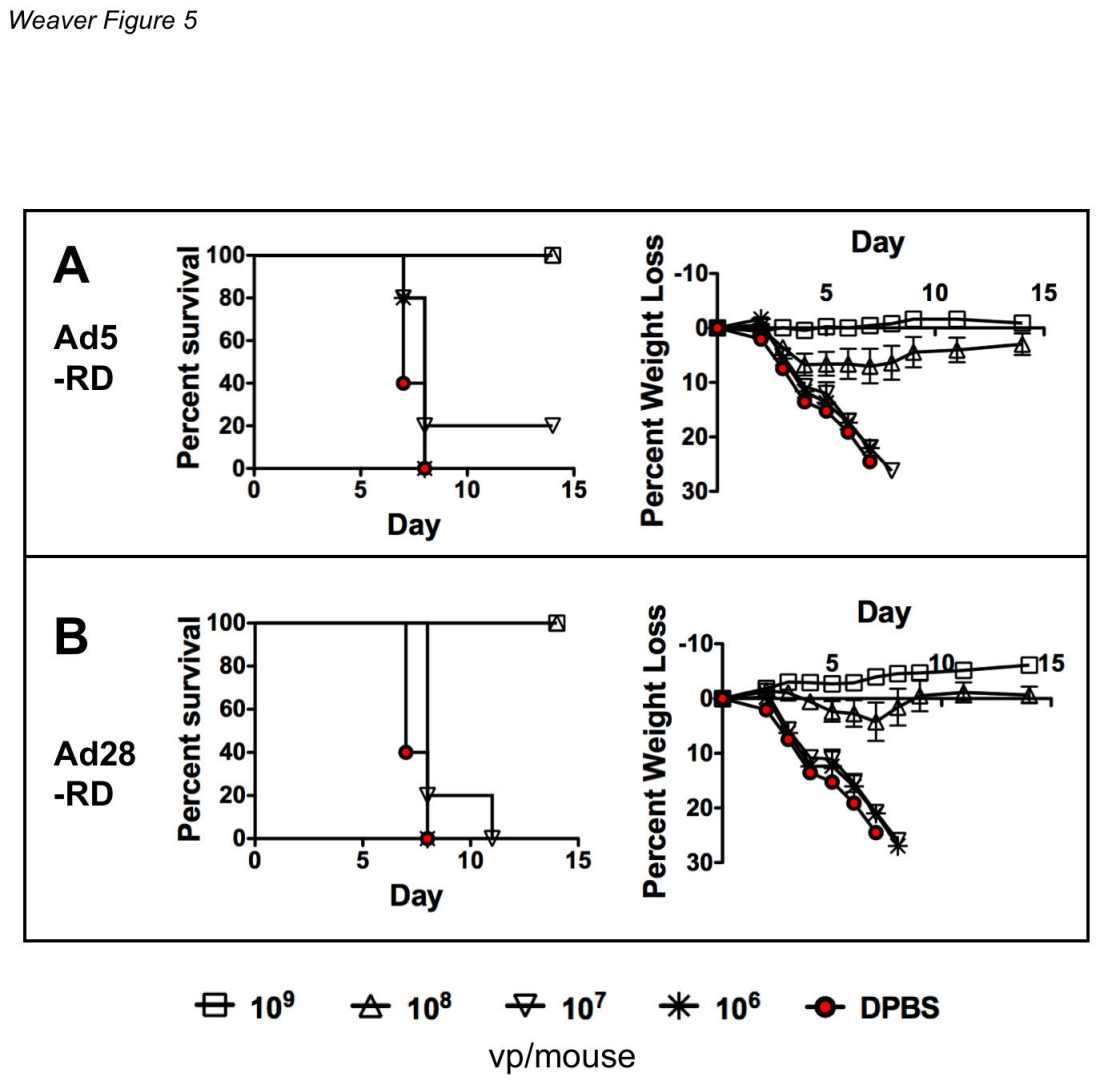
Dose titration of species C and D replication-defective Ad vectored vaccines. Groups of 5 BALB/c mice were immunized intranasally with 10-fold dilutions of vaccine from 10^9^ vp/mouse to 10^6^ vp/mouse. All viral vectors contained a transgene cassette that expressed a soluble hemagglutinin (HA) derived from the influenza strain A/PR/8/34 in place of the E1 genes. Mice were immunized with either replication-defective species C Ad5-RD (A) or replication-defective species D Ad28-RD (B). Three weeks after immunization the mice were anesthetized i.p. with ketamine (140 mg/kg)/xylazine (5.55 mg/kg). The mice were weighed for baseline measurements. Included in the sacrifice criteria was 25% body weight loss. The mice were challenged intranasally with 100MLD_50_ of influenza A/PR/8/34.

The replication-competent vaccines have the potential to replicate their genomes up to 10,000-fold to amplify antigen expression. To compare these to more traditional replication-defective Ad vaccines, replication defective Ad28 (Ad28-RD) and replication-defective Ad5 (Ad5-RD) expressing the same hemagglutinin were tested by the intranasal route (Figure 5). With these vectors, mice that were immunized with 10^8^ vp/mouse of Ad28-RD showed some weight loss. At 10^8^ vp/mouse, Ad5-RD (Figure 5A) showed more weight loss than Ad28-RD (Figure 5B). There was no protection in either group vaccinated with a dose of 10^6^ vp/mouse.

### Vaccine Efficacy in the Presence of Pre-existing Immunity to Adenovirus

**Figure 6:**
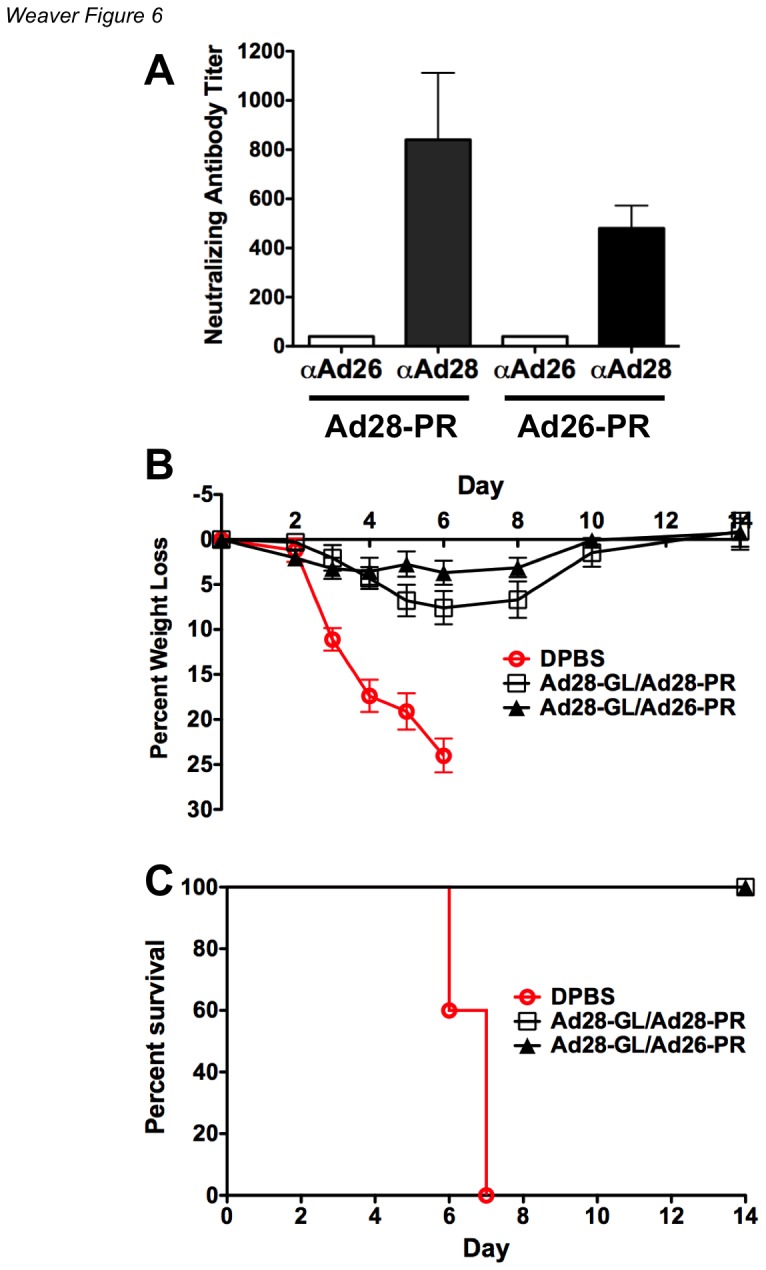
Vaccination in the presence of Anti-Vector Immunity. Pre-exisiting immunity was established by immunizing mice with Ad28 expressing GFPLuc. The sera was collected 6 weeks later and analyzed for anti-Ad26 and anti-Ad28 neutralizing antibodies (A). The mice were divided into two groups of 5 mice and immunized with either Ad26-PR or Ad28-PR influenza vaccines expressing the A/PR/8/34 HA. The mice were challenged intranasally with 100MLD_50_ of influenza A/PR/8/34. The mice were monitored for signs of disease and weighed daily (B). The mice were euthanized if the percent weight loss exceeded 25% of the baseline level (C).

Many vaccines need to be delivered at least two times for priming and boosting. To model this situation, mice were immunized intramuscularly with 10^10^ vp/mouse of Ad28 expressing GFPLuc. Sera were collected 6 weeks after immunization and tested for anti-Ad26 and anti-Ad28 neutralizing antibodies (Figure 6A). All immunized mice had high levels of Ad28 neutralizing antibodies (NAbs) but undetectable levels of Ad26 NAb. The mice were then vaccinated against influenza by intranasal administration of 10^10^ vp/mouse of Ad28-PR or Ad26-PR expressing the influenza A/PR/8/34 HA (Figure 6B and C). The mice were challenged intranasally with 100MLD_50_ of influenza A/PR/8/34 three weeks after the second immunization. In the face of prior Ad28 immunity, mice that were immunized with either Ad26-PR or Ad28-PR suffered slight weight loss (Figure 6B), but all survived the challenge (Figure 6C).

### Vector-Induced Innate Immunity

**Figure 7:**
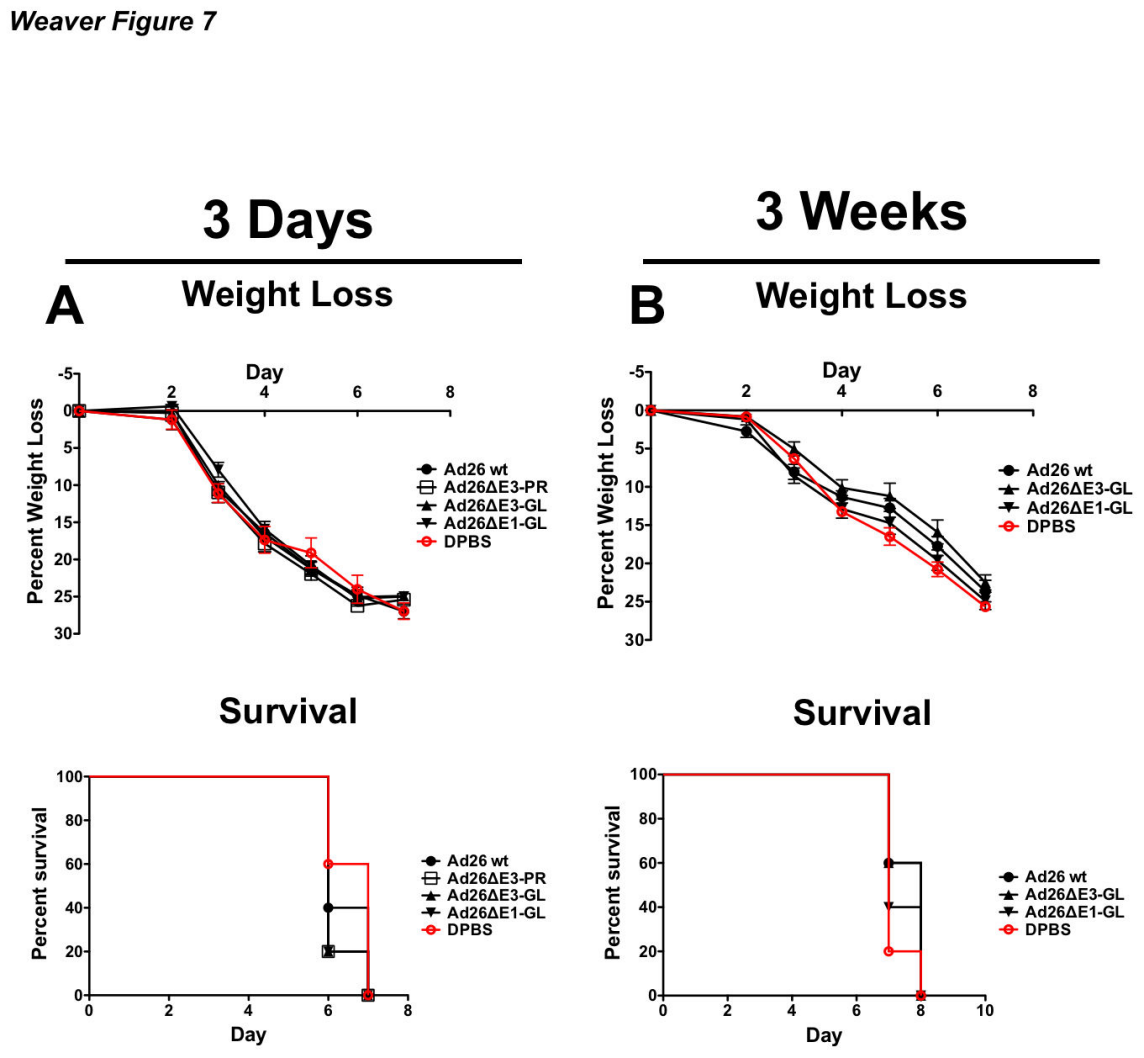
Induction of protection by innate immunity. Groups of 5 BALB/c mice were immunized intranasally with 10^10^ vp of wildtype Ad26 (Ad26 wt), replication-competent Ad26 expressing the PR HA or the eGFPLuc fusion gene (Ad26ΔE3-PR and Ad26ΔE3-GL, respectively) and a replication-defective Ad26 expressing the eGFPLuc fusion gene (Ad26ΔE1-GL). In order to identify vector-induced early non-specific innate immunity, the mice were challenged 3 days post-immunisation with 100MLD_50_ of influenza A/PR/8/34 (A). In order to identify vector-induced late non-specific innate immunity, the mice were challenged 3 weeks post-immunisation with 100MLD_50_ of influenza A/PR/8/34 (B). The mice were weighed for baseline measurements. Included in the sacrifice criteria was 25% body weight loss. All immunized mice had significant weight loss and mortality regardless of the expressed transgene or vector platform. There were no statistically significant differences in between groups or control DPBS mice at either time point.

Some studies have shown that innate immunity to empty Ad5-ΔE1/E3 virus could confer non-specific protection against influenza by innate immunity [30]. In order to rule out protection by innate immunity, groups of 5 mice were immunized intranasally with 10^10^ vp of Ad26 expressing the vaccine transgene or an irrelevant transgene (GL). Groups of mice were challenged with 100 MLD_50_ of influenza A/PR/8/34 3 days and 3 weeks post-immunization with vectors expressing GFPLuc. When mice were challenged 3 days post-immunization there was no change in weight loss and survival as compared to the control group (Figure 7A). Mice that were challenged 3 weeks post-immuization also showed significant weight loss and mortality as compared to the control group (Figure 7B). Ultimately, there were no differences in weight loss or survival in the immunized groups regardless of transgene, as compared to control DPBS immunized mice (Figure 7) Therefore, it is highly unlikely that innate immunity plays any role in the protection provided by species D Ad vectored vaccines. In addition, mice immunized with Ad26-PR and challenged at 3 days post-immunization were not protected against a lethal influenza challenge. However, mice that were challenged at 3 weeks post-immunization were protected (Figure 3). This suggests that a resting period is a necessity for the maturation of a protective immune response.

## Discussion

The philosophy of influenza virus control by vaccination has recently come under high levels of scrutiny. There have been numerous mismatches in vaccine formulations in the last decade that resulted in a lack of protective immunity. Influenza surveillance in Asia and Australia is used to predict which influenza strains might spread in the USA. While this is generally effective, these predictions can fail [4,31,32]. In fact, the accuracy of WHO recommended strains and those strains causing outbreaks was only 77% between 1987 and 1997 [33]. Put another way, influenza strain predications have failed 23% of the time leaving humans unprotected against seasonal and potentially pandemic influenza. The degree of vaccine mismatch from the 2009 pandemic Swine flu could not have been greater. The genetic distance between the vaccine strain and the pandemic strain was an impressive 20.5% based on the HA amino acid analysis. This is very alarming considering that there is only ~21% amino acid divergence in all of the H1 HA strains. It took 8 to 9 months for the first large-scale 2009 swine flu vaccine to become available and by that time the first wave of the pandemic influenza outbreaks had passed [34]. Most people would agree that this is unacceptable. In addition to the vaccine mismatches and failure to effectively control the recent 2009 swine flu pandemic, there is still the threat of “bird flu”, or H5N1 and H7N9. Scientists have published two research articles in which minor mutations in the HA gene could convert the bird flu to a form that was droplet transmissible in mammals [35,36]. The mortality rate in humans infected with H5N1 is greater than 50% and this coupled with the inevitability of future pandemics is plenty of cause for action to investigate alternatives to our current vaccine strategies.

In this study, we have demonstrated the strength of an Ad vectored vaccine. In a head to head comparison the Ad vaccine was capable of inducing cross-protective immunity while the traditional vaccine was not, even at a dose 375 times the human equivalent. This comparison indicates that Ad vectored vaccines may have much higher “take” rates than traditional vaccines, are capable of inducing cross-protective immunity and may be robust enough to induce protective immunity in the elderly and immunocompromised.

A dose titration of the species C and D vaccines showed similar results. In addition, there were no significant differences between the replication defective forms of species C and D vaccines. The inability of species D Ad vaccines to induce protective immunity when delivered intramuscularly can be explained by the lack of CD46 in mouse muscle. Species D Ads use human CD46 as the primary receptor for virus entry. Mice do not express this receptor. Therefore the lack of CD46 can account for the reduced transduction of the muscle. However, the D Ad vaccines were effective when delivered intranasally suggesting the presence of a functional mouse CD46 homolog or the use of a secondary receptor that is also not present in the muscle. We have found that species D Ad vectors are capable of transducing CD46 transgenic mice intramuscularly equally well as compared to the species C Ad5 vectors (data not shown). We propose that vaccine studies using these transgenic mice will provide a more accurate evaluation of their vaccine efficacy. If these Ad D vaccines are transducing via a secondary receptor intranasally, the restoration of the CD46 receptor may allow for even greater levels of transduction that results in the induction of protection at much lower doses than this study indicates.

The vaccine immunogen used in these studies, HA from A/PR/8/34, was modified to have a deletion of the cleavage, fusion and transmembrane domain (HA-ΔCFT). This was done in order to create a secreted protein that was resistant to proteolytic cleavage so that it would remain intact. We hypothesized that an intact secreted HA would induce greater humoral immunity. However, upon characterization we found that the full-length version was capable of generating protective immune responses at a lower dose. Dose response studies using Ad5 expressing the full-length A/PR/8/34 gene showed complete protection at 10^7^ vp/mouse [28]. However, the soluble form of HA required a dose of 10^9^ vp/mouse for complete protection against morbidity.

In order to determine if a species D Ad vaccine could be effective in the presence of pre-existing immunity, we established anti-Ad neutralizing antibodies by immunizing with Ad28 expressing GFPLuc. We tested the sera at 6 weeks post-immunization and found high anti-Ad28 neutralizing antibodies. We found that mice that had high levels of pre-existing immunity against Ad28 could still be protected against a lethal influenza challenge using a homologous Ad28-PR vaccine. This is promising since it demonstrates an effective ability to reuse the same vaccine even in the presence of high anti-vector immunity. One caveat to this observation is that the second immunization with the homologous Ad28-PR vaccine was at a high dose of 10^10^ vp/mouse. This dose may be too much to be effectively translated to a human dose equivalent. Further studies will determine if the vaccine dose could be titrated down to a more usable dose.

In this study we have investigated the use of species D Ad viral vectors as vaccines for influenza. One of the biggest advantages of using these viral vectors over other Ad species is that they have a low seroprevalence in humans. In addition, they exhibit low levels of pathogenicity, can be isolated from asymptomatic patients, and are not associated with any known severe disease. Importantly, we have shown that the route of immunization is critical when using species D Ad vaccines. The species D vaccines were more than 100-fold more effective when delivered mucosally. This is an important find since other researchers are investigating the use of species D Ad vaccines as well as other non-human primate Ad vaccines for use against HIV [37–41]. In addition, cloning the entire viral genomes into a single plasmid allows us to rapidly create recombinant viruses. We propose that, due to the findings in this study, these viral vectored vaccines could be an important alternative to the currently used TIV and FluMist vaccines and warrant further investigation.

## Materials and Methods

### Ethics Statement

Female BALB/c mice (6-8 weeks old) were purchased from Charles River Laboratories (Wilmington, Massachusetts, USA) and housed in the Mayo Clinic Animal Facility under the Association for Assessment and Accreditation of Laboratory Animal Care (AALAC) guidelines with animal use protocols approved by the Mayo Clinic Animal Use and Care Committee (protocol A110). All animal experiments were carried out according to the provisions of the Animal Welfare Act, PHS Animal Welfare Policy, the principles of the NIH Guide for the Care and Use of Laboratory Animals, and the policies and procedures of Mayo Clinic.

### Adenovirus vaccine production

In order to create new recombinant adenoviral vectors the genomes of Ad6, Ad26, Ad28 and Ad48 were cloned individually into a single low copy plasmid. The Ad5 vaccine was generated using the stratagene shuttle plasmids as previously described [42]. The E1 or E3 genes of the cloned adenoviral vectors were replaced with an expression cassette that expressed a hemagglutinin (HA) from A/PR/8/34 that was modified to have a deletion of the cleavage, fusion and transmembrane domain (HA-ΔCFT). Replication competent Ad vaccines were generated by replacing the E3 genes and replication defective versions were made by replacing the E1 genes. All of the viruses were rescued and amplified in 293 cells. Cell factories (Corning, Rochester, NY, USA) were used for large-scale preparations. The final virus preps were purified on two sequential CsCl ultracentrifuge gradients, desalted using Econopac® 10-DG chromatography columns (Bio-Rad, Herculaes, Ca, USA), resuspended in KPBS (1.0 M Sucrose) and stored at -80°C. The final concentrations were determined by OD260.

### Wild-type Adenovirus and gDNA purification

Wild-type (wt) Ad6 strain Tonsil 99 (ATCC VR-1083), Ad26 strain BP-2 (ATCC VR-224), Ad28 strain BP-5 (ATCC VR-226), and Ad48 strain T85-884 (ATCC VR-1406) (American Type Culture Collection, Manasses, VA, USA) were amplified in low passage 293 cells (Microbix Biosystems Inc., Toronto, Ontario, Canada). Viral genomic DNA (gDNA) was purified using a PureLink™ Viral RNA/DNA Mini Kit (Invitrogen, Carlsbad, CA, USA).

### Generation of Species C Adenoviral Type 6 Vector

The left and right arms of the Ad6 gDNA were amplified by PCR using Platinum Taq Supermix High Fidelity (Invitrogen, Carlsbad, CA, USA). A unique SgfI restriction enzyme site was added just outside of the left and right ITRs. The following primers were used to amplify the PCR product: Ad6 SgfI ITR (5’-GCGATCGCCATCATCAATAATATACCTTATTTTGGATTG-3’), Ad6 Cla1 R (5’- GGGGGCTGTAATGTTGTCTCTACGCCTGCACATAATCTAACACAAACTCCTCACCCTC-3’) and Ad6 AvrII F (5’- GAGGGTGAGGAGTTTGTGTTAGATTATGTGCAGGCGTAGAGACAACATTACAGCCCCC-3’). The 3’ end of Ad6 Cla1 R contained 28 nucleotides (nt) of homology to the right arm of the Ad6 gDNA from nt 35,208 to 35,235 and the 5’ end of Ad6 AvrII F contained 30 nt of homology to the left arm of the Ad6 gDNA from nt 979 to 1,008 (Figure S1A). The two PCR products were then joined using an overlapping PCR reaction. One µl of each PCR product and 2 µl of 10µM primer Ad6 SgfI ITR were added to 45 µl of Platinum Taq Supermix High Fidelity and extended (Figure S1B). The overlapping PCR product was cloned into TOPO PCR 2.1 (Invitrogen, Carlsbad, CA, USA). The Kanamycin resistance gene (KAN^R^) and the pBR322 origin of replication (pBR322 ori) from Ad-Easy (Stratagene) was PCR amplified using Platinum Taq Supermix High Fidelity and cloned into TOPO-XL (Invitrogen, Carlsbad, CA, USA) using the following primers: Kan ori SgfI-F (5’- GCGATCGCTAATTAACATGCATGGATCCATATGCGGTGTG-3’) and Kan ori SgfI-R (5’- GCGATCGCTTAATTAAGAATTAATTCGATCCTGAATGGCGAATGG-3’) (Figure S1C). The Kan^R^ and pBR322 ori were ligated to the overlapping PCR using PvuI sites to form the cloning plasmid, pAd6-cloner (Figure S1D). Ad6 gDNA and pAd6-cloner were digested with ClaI and AvrII, separated on a TAE agarose gel containing 17.5 µg/ml Crystal Violet, and purified using a S.N.A.P.™ Gel Purification Kit (Invitrogen, Carlsbad, CA, USA). The ClaI/AvrII gDNA fragment (Figure S1E) and the ClaI/AvrII digested pAd6-cloner were ligated to form the pAd6 ClaI/AvrII plasmid (Figure S1F). Finally, the AvrII digested gDNA fragment was ligated into the pAd6 ClaI/AvrII plasmid that was digested and purified as previously described to form the final construct, pAd6 (Figure S1G). The final construct was checked by RE digestion to confirm correct orientation, digested with SgfI, and desalted using a QIAexII purification kit (Qiagen, Valencia, CA, USA). The SgfI digested pAd6 was transfected into 293 cells using Polyfect (Qiagen, Valencia, CA, USA) and wt Ad6 virus was rescued.

### Generation of Species D Adenoviral Vectors

We have cloned the genomes of three species D Ads types 26, 28 and 48 into single low copy plasmids for use in creating recombinant adenoviruses. All of the viral genomes were cloned using similar strategies as previously described for Ad6. Briefly, for Ad26, two overlapping PCR products that were designed to extend into the 5’ and 3’ ends of the genome. The PCR products extended to the EcoRI sites (Figure S2A). To clone the Ad26 viral genome the following primers were used to amplify the right and left sides of the Ad26 genome with overlapping homology designed into the primers: Ad26 ITR-PacI 5’-ATTTAATTAACATCATCAATAATATACCCCACAAAGTAAACAAAAG, Ad26-Clone Frag-F 5’-AATGAGAGCAGGAGTGATGAATATGAATTCTATTACCATCTTCGTCCCTTAGCAA, Ad26-Clone Frag-R 5’-TTGCTAAGGGACGAAGATGGTAATAGAATTCATATTCATCACTCCTGCTCTCATT. The two overlapping PCR products were used to create a final full-length PCR product that fused the two PCR fragments together at the EcoRI site (Figure S2A). The primers used to create the fused PCR product were also designed to contain unique PacI 5’ restriction sites so that the captured genomes can be released from the plasmid for rescue in mammalian cells (Figure S2B). The fused overlap PCR products were cloned into TOPO-pCR2.1-TOPO (Invitrogen, Carlsbad, CA) and verified by sequencing. Next, the a plasmid backbone containing a low copy origin of replication (pBR322) and kanamycin resistance gene was PCR amplified using primers that contain 5’ and 3’ PacI restriction sites (Figure S2C). Figure S2D shows the final cloning plasmid. The Ad26 genomic DNA and the cloning plasmid were digested with EcoRI (Figure S2E). The cloning plasmid was dephosphorylated with Antarctic phosphatase and gel purified. The digested gDNA and cloning plasmid were mixed at a 3:1 insert to vector ratio and ligated overnight at 4C. The ligation mixture was used to transform electrocompent XL-1 blue cells and colonies were selected on Kanamycin LB plates. Miniprepped DNA was digested to confirm the capture of the EcoRI Ad26 gDNA in the correct orientation (Figure S2F). For Ad28, the unique restriction enzyme site EcoRI and NheI were used to capture the genome (Figure S3A and E). To clone the Ad28 genome the following primers were used to amplify and fuse the right and left side of the Ad28 genome: Ad28 F 5’- TGGGGTGCTGTTCATGGCCAACAGCCAAAGCTAGCACCCAAAAACTGCACGCT, Ad28 R 5’-AGCGTGCAGTTTTTGGGTGCTAGCTTTGGCTGTTGGCCATGAACAGCACCCCA and Ad28 ITR PacI 5’-TTAATTAACATCATCAATAATATACCCCAC. Figure S3F shows the final cloned Ad28 gDNA plasmid. For Ad48, the EcoRI and MluI sites were used to capture the genome (Figure S4A and E). To clone the Ad48 genome the following primers were used to amplify and fuse the right and left side of the Ad48 genome: Ad48 PacI F1 5’-ATTTAATTAACATCATCAATAATATACCCCACAAAGTAAACAAAAGTTAATATGC, Ad48 R 5 prime 5’-GCGTAGAGCCACTGCTCGAAGCCACAGATGAAGATCATGGAATTCATATTCATCA, and Ad48 F 3 prime 5’-TGATGAATATGAATTCCATGATCTTCATCTGTGGCTTCGAGCAGTGGCTCTACGC. Figure S4F shows the final cloned Ad48 gDNA plamid.

### Recombinant Adenoviral Vectors

Shuttle plasmids were generated to transfer genes into each of the cloned adenovirus genomes by recombination in bacteria (Figure S5). These shuttle plasmids are composed of an expression cassette containing a secretable intact HA gene driven by the cytomegalovirus enhancer/promoter that is adjacent to the zeocin antibiotic resistance marker. Both the transgene and selective marker are flanked by homologous sequence of the adenovirus region that will be recombined. The following briefly describes how the shuttles were constructed. First, primers were designed to produce an overlapping PCR product containing a unique AscI site between the overlapping products (Figure S5A and C). The overlapping PCR products were designed to amplify ~500 nucleotides of the regions outside the area specified for recombination. The two PCR reactions were combined and extended to produce a fused PCR product containing a unique AscI site between the two homology regions flanked by PmeI sites (Figure S5B and C). The transgene was prepared by cloning the transgene into a CMV-PolyA expression cassette (e.g. CMV-GFPLuc-PolyA). The selection marker containing the EM7 promoter and zeocin resistance gene gene were amplified with homology to the transgene expression cassette 5’ to the PolyA and recombined into the plasmid using the red recombinase system [43]. The zeocin gene was flanked by FRT sites to facilitate removal by FLP recombinase at a later time point if needed (Figure S5D). Next, the entire expression cassette with selection marker was amplified using primers with AscI sites engineered into the 5’ and 3’ ends. This PCR product was cloned into the Topo pCR8 cloning plasmid to create the plasmid pCR8-AscI-GFPLuc-FZF-AscI. The plasmid was sequence verified. The final shuttle plasmid for recombination into the adenoviral genome was created by cloning the AscI digested insert from pCR8-cdE3-AscI-GFPLuc-FZF-AscI into the plasmid containing the overlapping PCR product at the AscI sites (Figure S5E). These shuttle plasmids were digested with PmeI (Figure S6A) and recombined into the complete Ad genome plasmid using in vivo recombination in BJ5183 competent cells (Figure S6B) to generate the final vector (e.g. pAd26-dcE3-PR-dCFT-FZF plasmid) (Figure S6C). The final recombined plasmids were subcloned in XL-1 cells and plasmid DNA was amplified and purified using a Qiagen HiSpeed Maxiprep kit.

The modified viral genomes were digested from their plasmid backbones using PacI and transfected into 293 cells. The rescued virus was amplified and purified on two sequential CsCl gradients.

### Adenovirus immunization

Mice were anesthetized by intraperitoneal (i.p.) injection with ketamine (140 mg/kg)/kylazine (5.55 mg/kg) and were immunized by intramuscular (i.m.) or intranasal (i.n.) routes. Mice immunized by the i.m. route received the Ad vaccine or the monovalent inactivated virus (MIV) H1N1 2009 (BEI Resources) in two 25 µl injections into each quadricep muscle (50 µl total volume). Mice immunized by the i.n. route received the Ad vaccine in two 10 microliter instillations into each nare (20 µl total volume).

### Hemaglutination Inhibition (HII) Assay

The HI assay was performed as previously described [28]. Briefly, Groups of 5 BALB/c mice were immunized i.m. and i.n. with 10^10^ vp/mouse of adenovirus expressing the HA-∆CFT codon-optimized gene from influenza A/PR/8/34. The mice were bled three weeks post-immunization and serum was separated using Becton Dickinson microtainer tubes. Two-fold dilutions of sera in 50 microliters of DPBS were added to a 96-well, nonsterile, nontissue culture–treated, round bottom microtiter plate. Four hemagglutinating units (HAU) of influenza virus in 50 µl was added to the diluted sera and incubated at room temperature (RT). After 1 hr., 50 µl of a 1.0% chicken RBC solution was added and incubated at RT for 1 hr. The HI titer was determined to be the highest serum dilution to inhibit hemagglutination.

### Influenza challenge

Mice were challenged intranasally three weeks after immunization. The mice were anesthetized and challenged with 100 MLD_50_ (50% mouse lethal doses) of mouse adapted influenza virus A/PR/8/34 by two 10 microliter instillations into each nare (20 µl total volume). The mice were observed for morbidity and weight loss over subsequent days and were euthanized if weight loss reached 25% of initial body weight.

### Adenovirus Neutralization Assay

Groups of mice were immunized intramuscularly with 10^10^ vp/mouse of Ad28 expressing the eGFP/Luciferase fusion protein. Six weeks post immunization the mice were bled by the submandibular method and the sera was tested for anti-adenovirus neutralizing antibodies (NAbs). NAbs were determined as previously described [44]. Briefly, sera were heat inactivated at 56°C for 30 min before a serial 2-fold dilution was performed in a 96 well black plate (3603 Corning). Sera were diluted from 1/20 to 1/2560 in a volume of 50 µl of cDMEM containing 1 X 10^8^ Ad-GL vp/ml. Naïve mouse serum was used as negative controls and was the maximum luciferase activity reference. The plates were incubated at 37°C for 1 h. Fifty µl of A549 cells at 1 X 10^5^ cells/ml was added to each well and the plates were incubated for 20 h at 37°C and 5% CO_2_ before readout. The luciferase activity was determined using the reporter lysis 5X buffer and luciferase assay reagent (LAR) (Promega, Madison, WI) as described by the manufacturer protocol. Briefly, 25 µl of 5X lysis buffer was added to each well, mixed and frozen at -80C. The plates were thawed at RT and 50 µl of LAR was added. The plate was shaken on an orbital shaker and luciferase activity was measured using a Beckman Coulter DTX 880 Multimode Detector. Neutralizing antibodies titers were determined as the reciprocal dilution to inhibit 50% of luciferase activity as compared to no serum controls and are expressed as the geometric mean titer (GMT).

### Statistical Analyses

Data was evaluated using GraphPad Prism 4 software. Unpaired, two-tailed TTests, ANOVA with Bonferroni post test and Log-rank (Mantel-Cox) tests were used to determine statistical significance. P values ≤ 0.05 were considered statistically significant.

## Supporting Information

Figure S1 **The cloning strategy for Adenovirus type 6.**
An overlapping PCR product that fuses the left and right regions of the Ad6 genome at the ClaI and AvrII restriction sites (A and E). This PCR product was ligated to the low copy origin of replication and kanamycin resistance gene (C) to create the cloning plasmid (D). The genomic DNA of Ad6 is digested with Cla I and AvrII and ligated into the pAd26 cloner to create the pAd6 ClaI/AvrII plasmid (F). The AvrII genomic fragment was then ligated in to create the pAd6 gDNA correct plasmid (G).
(TIFF)

Figure S2 **The cloning strategy for Adenovirus type 26.**
An overlapping PCR product that fuses the left and right regions of the Ad26 genome at the unique EcoRI restriction site (A and E) was ligated to the low copy origin of replication and kanamycin resistance gene (C) to create the cloning plasmid (D). The genomic DNA of Ad26 is digested with EcoRI and ligated into the pAd26 cloner to create the plasmid pAd26 gDNA correct (F).
(TIFF)

Figure S3 **The cloning strategy for Adenovirus type 28.**
An overlapping PCR product that fuses the left and right regions of the Ad28 genome at the unique EcoRI and NheI restriction sites (A and E) was ligated to the low copy origin of replication and kanamycin resistance gene (C) to create the cloning plasmid (D). The genomic DNA of Ad28 was digested with EcoRI and NheI (E). The digested genomic DNA was ligated into the pAd28 cloner plasmid to create the plasmid pAd28 complete 4.5Kb kan ori (F).
(TIFF)

Figure S4 **The cloning strategy for Adenovirus type 48.**
An overlapping PCR product that fuses the left and right regions of the Ad48 genome at the unique EcoRI and MluI restriction sites (A and E) is ligated to the low copy origin of replication and kanamycin resistance gene (C) to create the cloning plasmid (D). The genomic DNA of Ad48 was digested with EcoRI and ligated into the pAd48 cloner to create the plasmid pAd48 gDNA correct (F).
(TIFF)

Figure S5 **Construction of shuttle plasmids to modify Adenoviral genomes.**
First, primers are designed to produce an overlapping PCR product that contains a unique AscI site between the overlapping products (A). The overlapping PCR products are designed to amplify the regions outside the area specified for recombination (C). To illustrate this design we used the Ad26 E3 deletion shuttle plasmid. 500 nucleotides of both the Ad26 17.5K and 14.7K gene were PCR amplified with primers with homology designed into the 3’ end of the 17.5K product and the 5’ end of the 14.7K product. The two PCR reactions were combined and extended to produce a fused PCR product (B). The final fused PCR product was designed to possess a unique AscI site between the two PCR product (B). The Ad26-dE3-PCR-AscI-PmeI PCR product was cloned into the Topo-pCR8 cloning plasmid and sequenced. The transgene was prepared by cloning the transgene (HA-∆CFT) into a CMV-PolyA expression cassette (D). To insert the selection marker, the EM7 promoter and zeocin gene were PCR amplified with homology to the transgene expression cassette 5’ to the PolyA. The zeocin gene was flanked by FRT sites to facilitate removal by FLP recombinase at a later time point if needed. Next the entire expression cassette and the selection marker was amplified using primers designed with AscI sites engineered into the 5’ and 3’ ends (D). The final plasmid, pCR8-Ad26-dE3-HA-dCFT-FZF was created by cloning the AscI flanked transgene into the shuttle plasmid for recombination into the E3 region.
(TIFF)

Figure S6 **Recombination into the Adenoviral gDNA.**
The shuttle plasmid was digested with PmeI (A) and cotransfected into BJ5183 cells to recombine into the complete Ad26 genome plasmid (B). The final recombined pAd26-dcE3 PR-dCFT-FZF plasmid was transformed into XL-1 cells and maxiprepped (C).
(TIFF)

Figure S7 **Analysis of HA expression by recombinant Adenoviral Vectors.**
293 and A549 cells were infected with 1000 vp/cell with each of the Ad-HA expressing viruses. The cells were harvested at 24h. Lysates from the infected cells were electrophoresed and transferred to PVDF membranes. After blocking, the membranes were probed with polyclonal anti-HA donkey sera (BEI Resources) and monoclonal anti-actin-HRP (I-19, Santa Cruz Biotechnology). The anti-HA donkey antibody was detected by rabbit anti-donkey-HRP (BioRad). After washing, the blot was developed using Super Signal West Dura substrate and imaged using a Kodak In Vivo F Imaging detector. Densitometry was performed using the Kodak Molecular Imaging Software v 4.0.4.
(TIFF)
