# Supplementary figures and images for: Low Seroprevalent Species D Adenovirus Vectors as Influenza Vaccines
Source: PLoS One. 2013 Aug 22;8(8):e73313. doi: 10.1371/journal.pone.0073313 (PMC3749993; doi:10.1371/journal.pone.0073313)

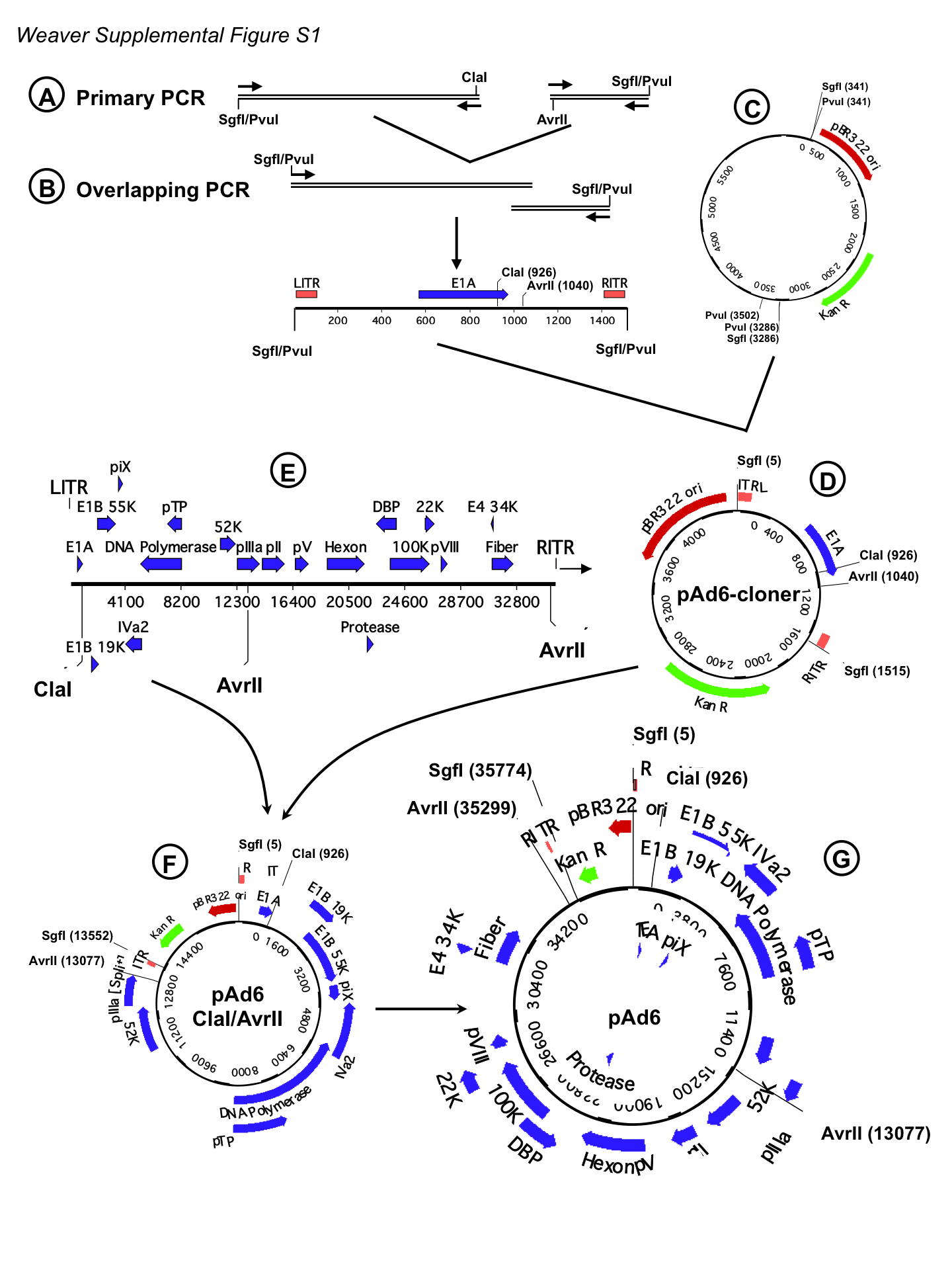

Supplement: Figure S1 — The cloning strategy for Adenovirus type 6. An overlapping PCR product that fuses the left and right regions of the Ad6 genome at the ClaI and AvrII restriction sites (A and E). This PCR product was ligated to the low copy origin of replication and kanamycin resistance gene (C) to create the cloning plasmid (D). The genomic DNA of Ad6 is digested with Cla I and AvrII and ligated into the pAd26 cloner to create the pAd6 ClaI/AvrII plasmid (F). The AvrII genomic fragment was then ligated in to create the pAd6 gDNA correct plasmid (G). (TIFF) [file pone.0073313.s001.tiff]

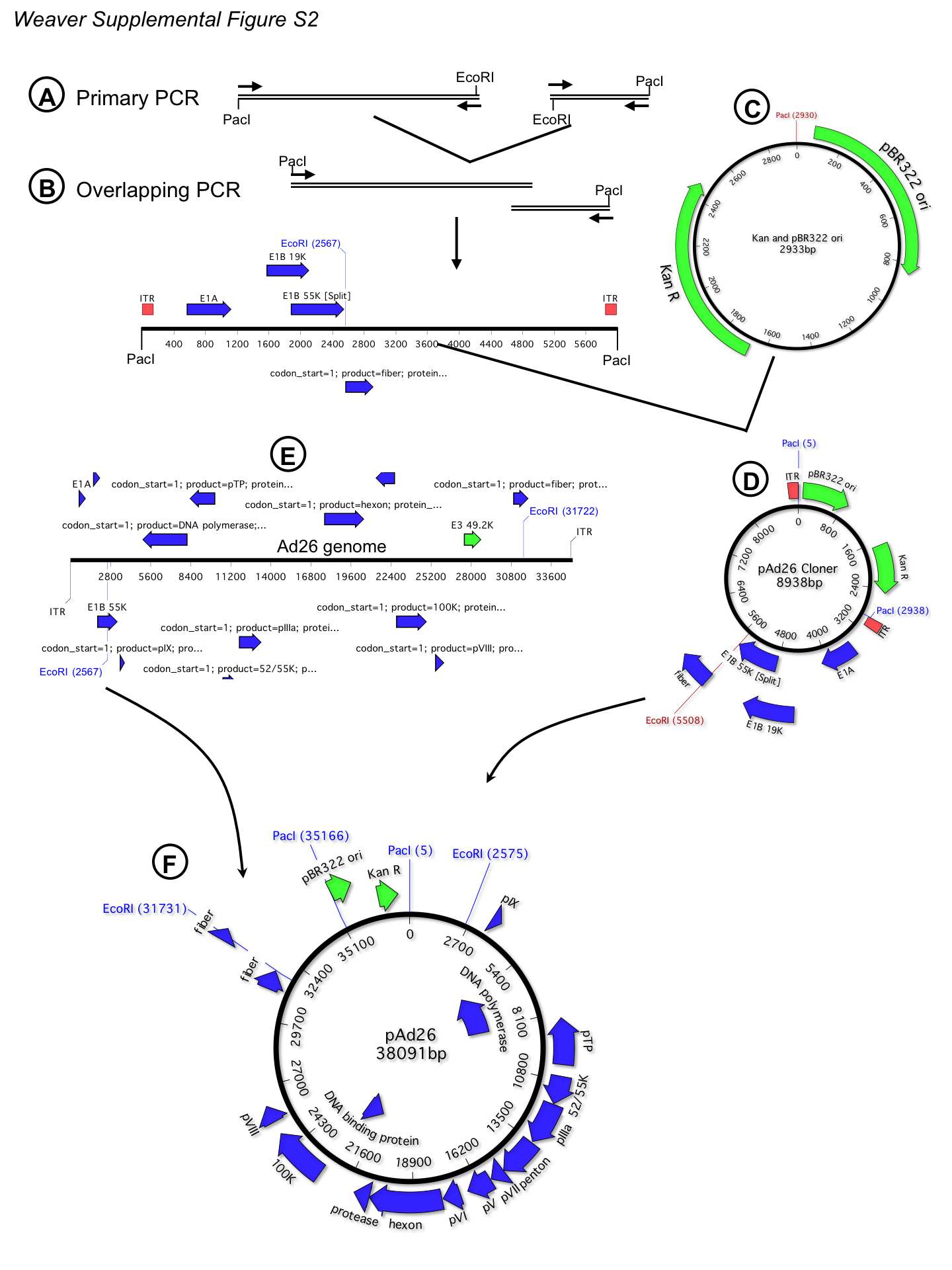

Supplement: Figure S2 — The cloning strategy for Adenovirus type 26. An overlapping PCR product that fuses the left and right regions of the Ad26 genome at the unique EcoRI restriction site (A and E) was ligated to the low copy origin of replication and kanamycin resistance gene (C) to create the cloning plasmid (D). The genomic DNA of Ad26 is digested with EcoRI and ligated into the pAd26 cloner to create the plasmid pAd26 gDNA correct (F). (TIFF) [file pone.0073313.s002.tiff]

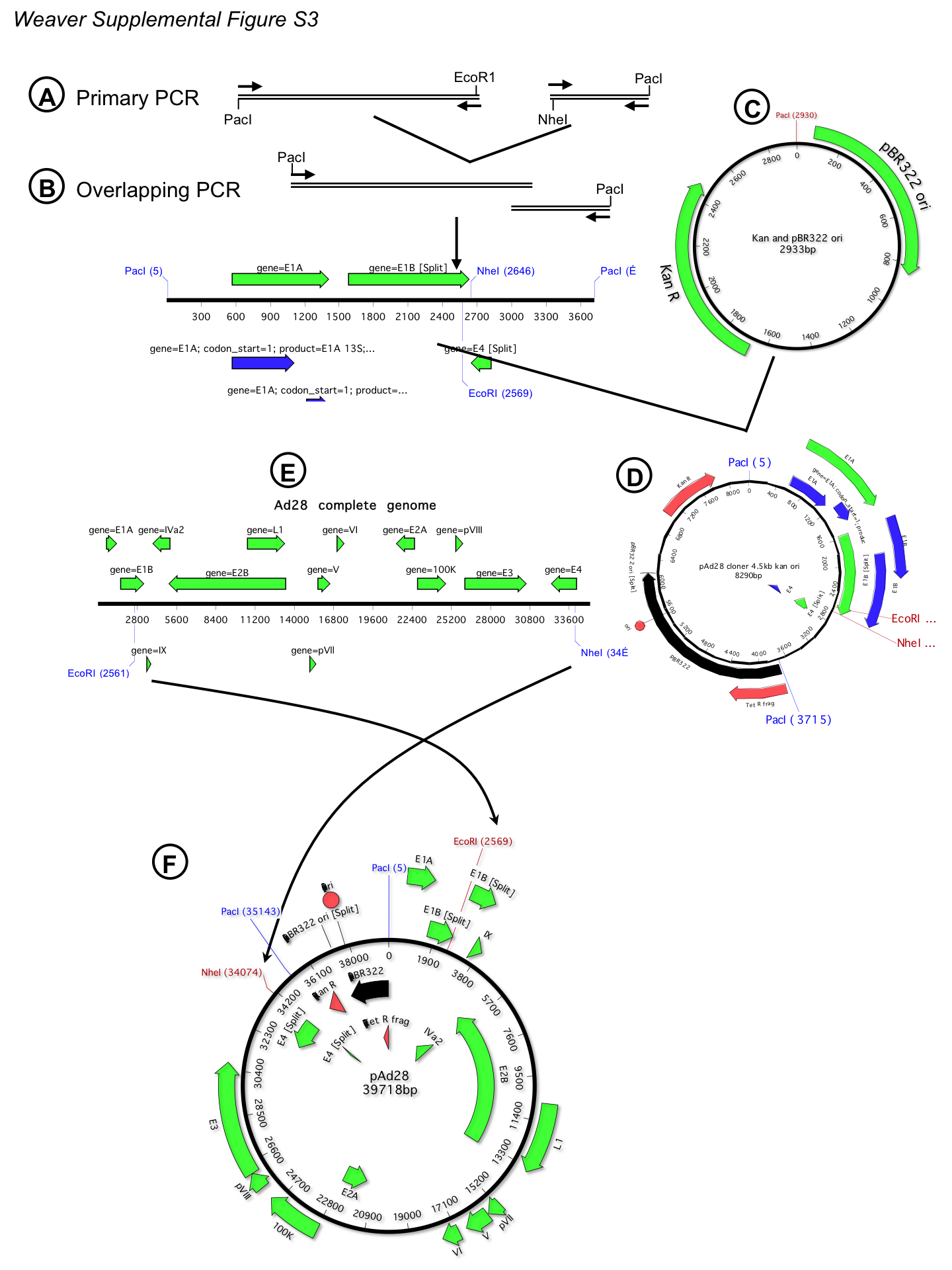

Supplement: Figure S3 — The cloning strategy for Adenovirus type 28. An overlapping PCR product that fuses the left and right regions of the Ad28 genome at the unique EcoRI and NheI restriction sites (A and E) was ligated to the low copy origin of replication and kanamycin resistance gene (C) to create the cloning plasmid (D). The genomic DNA of Ad28 was digested with EcoRI and NheI (E). The digested genomic DNA was ligated into the pAd28 cloner plasmid to create the plasmid pAd28 complete 4.5Kb kan ori (F). (TIFF) [file pone.0073313.s003.tiff]

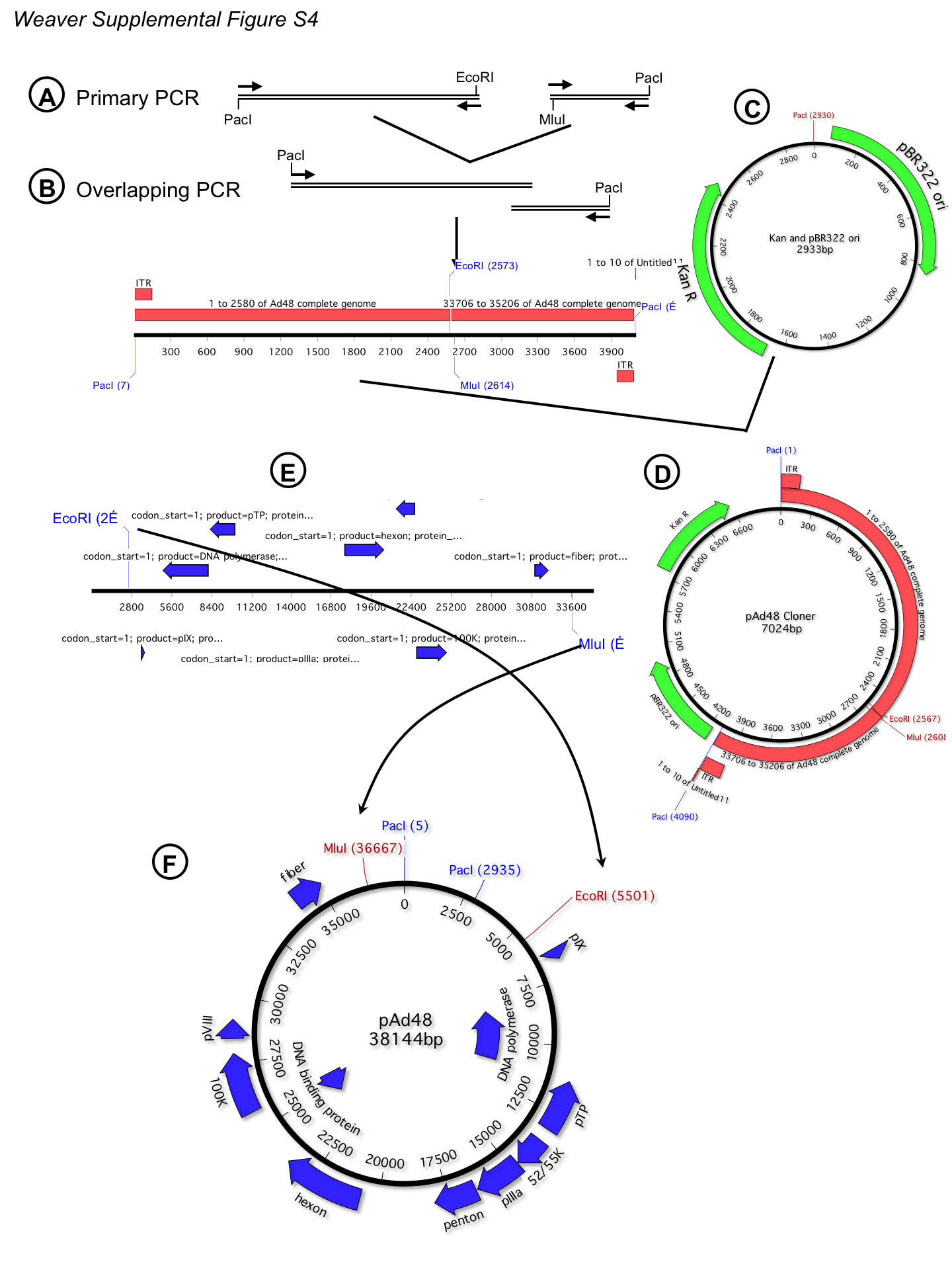

Supplement: Figure S4 — The cloning strategy for Adenovirus type 48. An overlapping PCR product that fuses the left and right regions of the Ad48 genome at the unique EcoRI and MluI restriction sites (A and E) is ligated to the low copy origin of replication and kanamycin resistance gene (C) to create the cloning plasmid (D). The genomic DNA of Ad48 was digested with EcoRI and ligated into the pAd48 cloner to create the plasmid pAd48 gDNA correct (F). (TIFF) [file pone.0073313.s004.tiff]

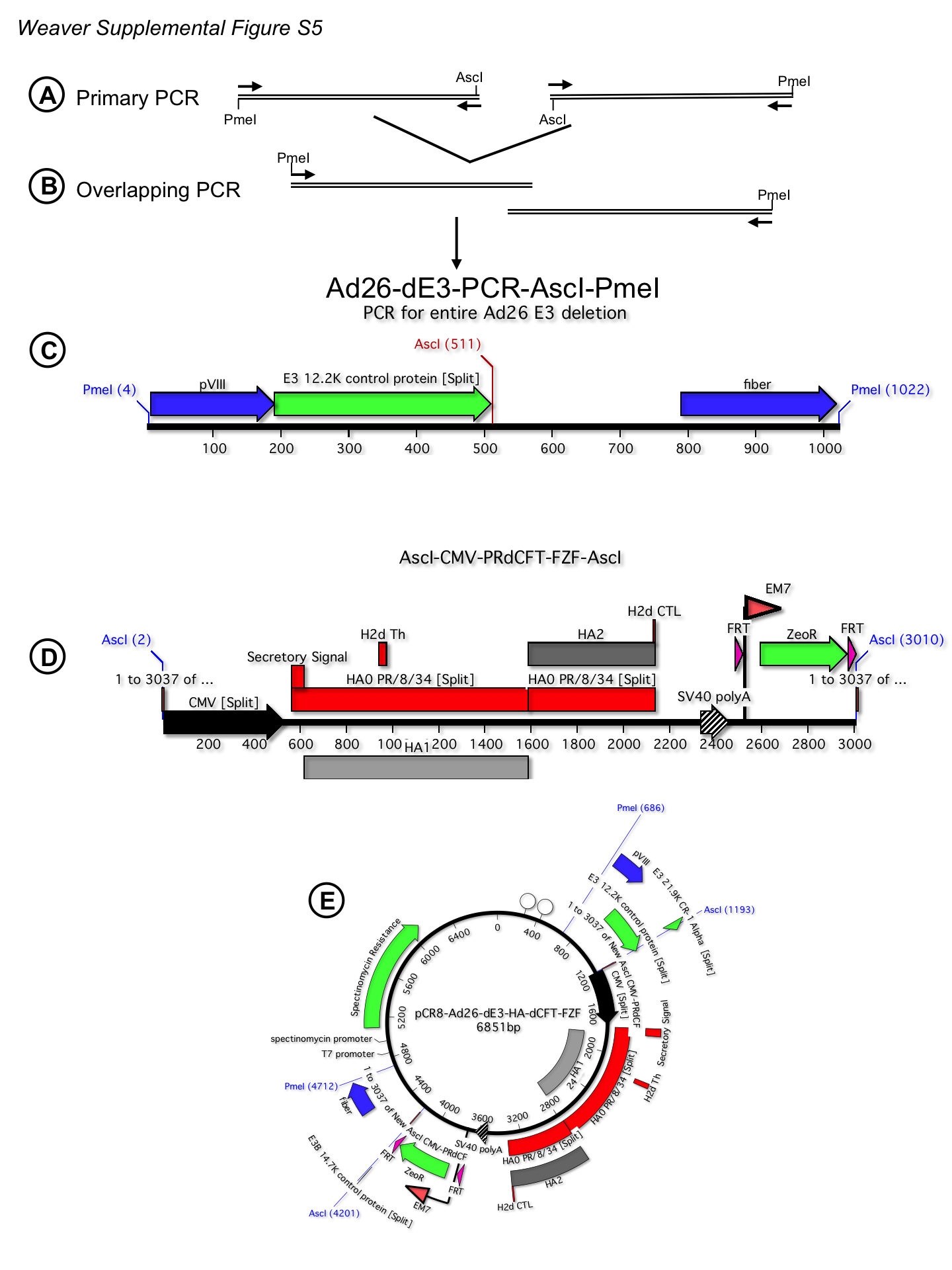

Supplement: Figure S5 — Construction of shuttle plasmids to modify Adenoviral genomes. First, primers are designed to produce an overlapping PCR product that contains a unique AscI site between the overlapping products (A). The overlapping PCR products are designed to amplify the regions outside the area specified for recombination (C). To illustrate this design we used the Ad26 E3 deletion shuttle plasmid. 500 nucleotides of both the Ad26 17.5K and 14.7K gene were PCR amplified with primers with homology designed into the 3’ end of the 17.5K product and the 5’ end of the 14.7K product. The two PCR reactions were combined and extended to produce a fused PCR product (B). The final fused PCR product was designed to possess a unique AscI site between the two PCR product (B). The Ad26-dE3-PCR-AscI-PmeI PCR product was cloned into the Topo-pCR8 cloning plasmid and sequenced. The transgene was prepared by cloning the transgene (HA-∆CFT) into a CMV-PolyA expression cassette (D). To insert the selection marker, the EM7 promoter and zeocin gene were PCR amplified with homology to the transgene expression cassette 5’ to the PolyA. The zeocin gene was flanked by FRT sites to facilitate removal by FLP recombinase at a later time point if needed. Next the entire expression cassette and the selection marker was amplified using primers designed with AscI sites engineered into the 5’ and 3’ ends (D). The final plasmid, pCR8-Ad26-dE3-HA-dCFT-FZF was created by cloning the AscI flanked transgene into the shuttle plasmid for recombination into the E3 region. (TIFF) [file pone.0073313.s005.tiff]

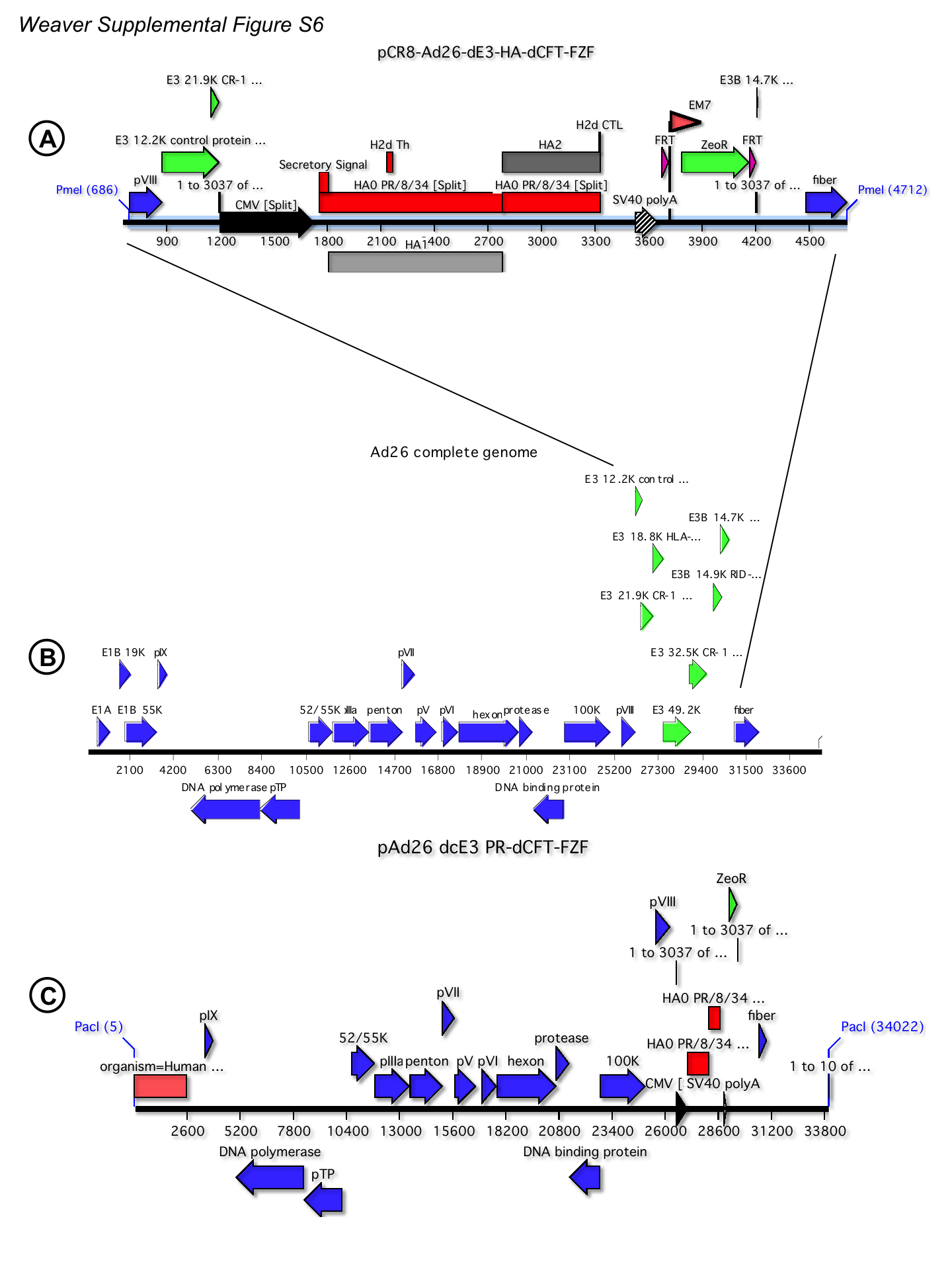

Supplement: Figure S6 — Recombination into the Adenoviral gDNA. The shuttle plasmid was digested with PmeI (A) and cotransfected into BJ5183 cells to recombine into the complete Ad26 genome plasmid (B). The final recombined pAd26-dcE3 PR-dCFT-FZF plasmid was transformed into XL-1 cells and maxiprepped (C). (TIFF) [file pone.0073313.s006.tiff]
